# Anti-VEGF in a Marathon Runner's Retinopathy Case

**DOI:** 10.1155/2016/5756970

**Published:** 2016-06-22

**Authors:** Alexander Kahjun Soon, Paulo Ricardo Chaves de Oliveira, David Robert Chow

**Affiliations:** ^1^McMaster University, Waterloo Regional Campus, Kitchener, ON, Canada; ^2^Toronto Retina Institute, North York, ON, Canada M3C 0G9; ^3^Department of Ophthalmology & Vision Sciences, University of Toronto, Toronto, ON, Canada M5B 1W8

## Abstract

Central retinal vein occlusion (CRVO) is one of the most common retinal vascular disorders. Intense exercise associated CRVO have been described in otherwise healthy young patients. We describe a case of a young male ultramarathoner who presented with a CRVO, presumably associated with dehydration, making part of a marathon runner's retinopathy. Resolution of macular edema and subretinal fluid, with visual acuity improvement, was observed after 3 monthly injections of ranibizumab. Our case suggests that dehydration could be involved in the mechanism of CRVO in healthy young patients and ranibizumab may be an effective treatment option for marathon runner's retinopathy.

## 1. Introduction

Central retinal vein occlusion (CRVO) is one of the most common retinal vascular disorders. It can be classified as ischemic and nonischemic, generally defined by the fluorescein angiography findings. It usually occurs in the elderly population, although younger subjects are still susceptible. Complications of CRVO include macular edema, ocular neovascularization, and neovascular glaucoma that can lead to low vision and blindness [1, 2].

Among the predisposing factors, aging, arterial hypertension, diabetes, and glaucoma play an important role. Also, especially in younger patients presenting with CRVO, other abnormalities, such as increased blood viscosity, protein C or S deficiency, vasculitis, hyperhomocysteinemia, and factor V Leiden mutation, should be investigated [2, 3].

Intense exercise associated CRVO, with possible alteration on blood rheologic factors, have been described in otherwise healthy young patients [4]. The term marathon runner's retinopathy was coined by Labriola et al. in 2009 [5]. We report a similar case of CRVO occurring in a highly trained athlete, following a complete marathon run, who was successfully treated with ranibizumab.

## 2. Case Report

A 24-year-old male marathon runner was referred to the Toronto Retina Institute for examination of suspected CRVO. The patient reported competing twelve marathons and eight half-marathons within the past two years. Over the course of two to three months, the patient reported a single, “purple” defect in the left lower quadrant, along with blurriness within the central vision in the right eye. He stated that his ocular symptoms closely followed a recent marathon that he completed in a state of severe dehydration due to his lack of hydration prior to and during the race and intermittent symptoms of presyncope. Further, symptoms progressed with peripheral vision loss in the right-inferior quadrant. Significant medical and social history included a two-pack-year smoking history.

**Figure 1:**
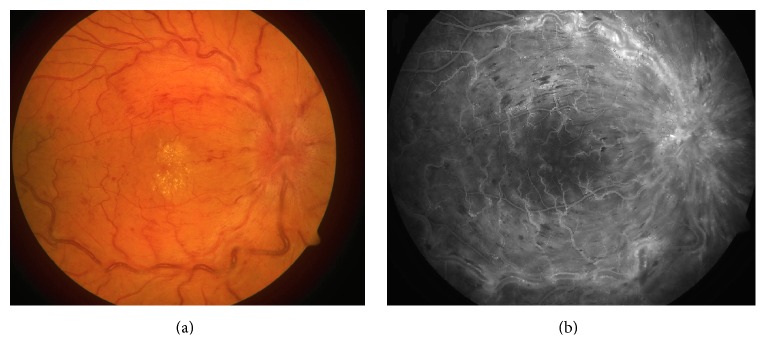
(a) Fundus retinography of the right eye (Canon CX-1, Canada): diffuse retinal and optic disc edema, venous dilation, and tortuosity; (b) intravenous fluorescein angiography showing nonischemic central retinal vein occlusion. Hemorrhagic blockage, venous tortuosity, and dilation, along with leakage from the optic disc and macula, are present.

**Figure 2:**
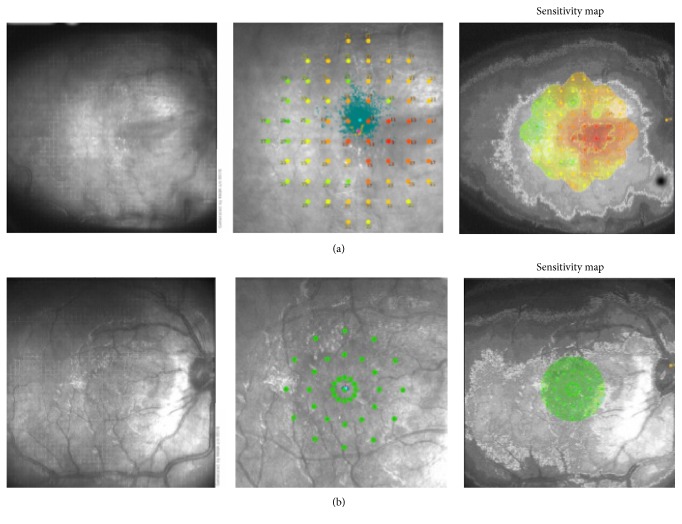
Fundus microperimetry (Macular Integrity Assessment, MAIA, CenterVue, Padova, Italy) of the right eye: initial exam, at presentation, showing decreased retinal sensitivity (a). Following ranibizumab treatment, marked improvement in retinal sensitivity (b).

**Figure 3:**
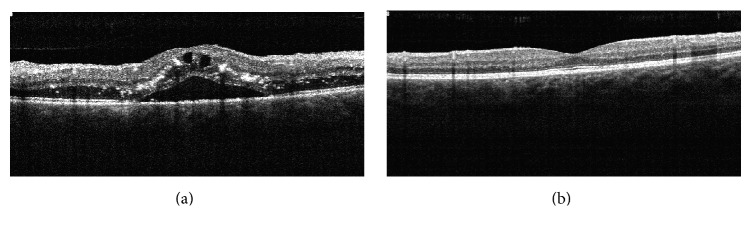
Optical coherence tomography (RTVue OCT, Optovue, California, USA) of the right eye: macular edema, with intraretinal cysts and subretinal fluid. Hyperreflective areas within the internal layers could correspond to hard exudates (a). Following treatment with ranibizumab, with complete resolution of the macular edema and neurosensory retinal detachment (b).

On eye exam, best-corrected visual acuity was 20/100 in the right eye and 20/20 in the left eye. Pupillary reflexes were within normal limits. Anterior surface slit-lamp examination was normal. Dilated fundus exam revealed diffuse, retinal, and optic disc edema, venous dilation, and tortuosity in all quadrants and multiple, perifoveal hard exudates (Figure 1(a)) in the right eye. Intravenous fluorescein angiography (IVFA) showed nonischemic central retinal vein occlusion (Figure 1(b)). Fundus microperimetry showed spots of evident decrease in retinal sensitivity (Figure 2(a)). Optical coherence tomography (OCT) of the right eye demonstrated marked macular edema (Figure 3(a)). The left eye was unremarkable and remained stable.

A complete medical evaluation, including systemic blood work, and hypercoagulability status were ordered, but the patient refused to do the exams due to financial issues.

**Figure 4:**
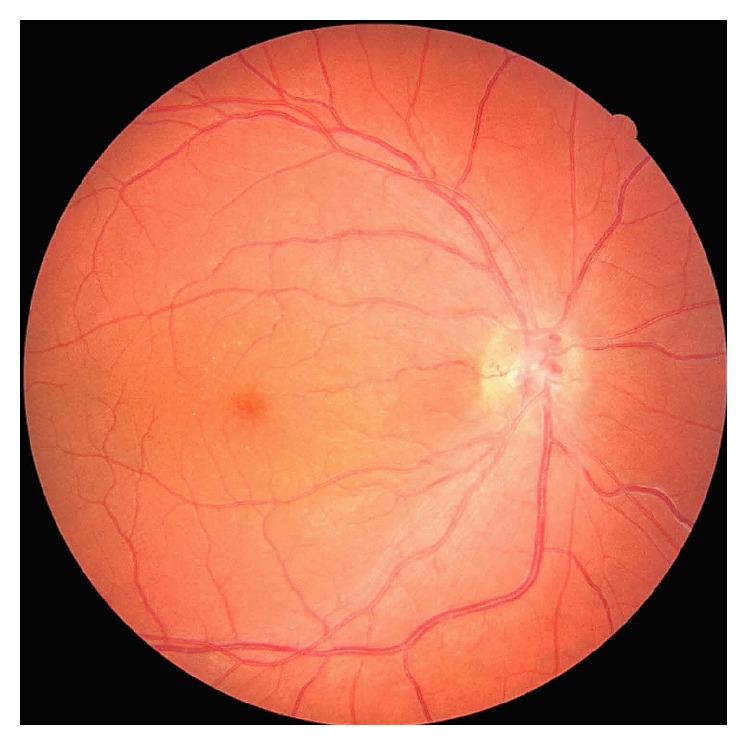
Fundus retinography (Canon CX-1, Canada) of the right eye, after ranibizumab treatment, showed resolution of retinal and optic disc edema, venous dilation, and tortuosity.

Treatment with intravitreal anti-vascular endothelial growth factor (anti-VEGF) was introduced. A total of six ranibizumab injections were administered over the course of eight months, following a treat-and-extend protocol. On the final assessment, his visual acuity was 20/20 in both eyes. Fundus alterations were resolved (Figure 4). Microperimetry (Figure 2(b)) and OCT (Figure 3(b)) showed improvements on the final visit.

## 3. Discussion

Young adults, under the age of 45, correspond to only about 13% of CRVO cases. In this context, hypercoagulability states, vasculitis, medications, trauma, hyperlipidemia, hyperhomocysteinemia, and other unusual causes should be ruled out. Intense exercise may be associated with CRVO in young patients, with no other comorbidities [4, 6, 7].

Extreme exercise, such as marathon running, might be accompanied by increase in hematocrit, plasma viscosity, and erythrocyte aggregability, especially when the subject loses a considerable amount of fluid, resulting in reduced blood flow and vascular occlusion susceptibility, although contrasting findings have been published [4, 7, 8]. Labriola et al. reported a similar case in a marathon runner and postulated that Virchow's triad, which involves damage to the blood vessel wall, changes in blood flow, and increase in coagulation factors, should be associated with the mechanism of CRVO in these individuals [5].

Our patient, a young man with no positive medical history, should have gone through a complete medical evaluation, in order to discard any associated condition, as earlier described. However, due to lack of health insurance coverage and financial conditions, he declined further investigation. Nevertheless, since the initial symptoms followed the marathon run, a presumptive diagnosis of CRVO secondary to intense exercise and severe dehydration was assumed.

The current treatment for macular edema in CRVO includes a variety of drugs, such as triamcinolone, dexamethasone, bevacizumab, ranibizumab, and aflibercept intraocular injections. However, in such patients, there is no consensus on which should be used, notably because of the small number of subjects and lack of randomized trials. Moisseiev et al. and Jacobs et al. showed improvement in visual acuity and resolution of macular edema, in similarly reported cases with the use of bevacizumab [7, 9]. In our patient, we decided to use ranibizumab, which produced an excellent result. Within 3 months of the first injection, the vision improved to 20/20 with total resolution of macular edema/subretinal fluid and no signs of ocular neovascularization, at which point we started to increase the week intervals between injections. This corroborates what the meta-analysis of six trials and 937 patients with macular edema from CRVO demonstrated: intravitreal anti-VEGF treatments increased the chance of significant gain in vision (at least 3 lines on the vision chart) when compared to no treatment [1].

This case report aims to raise awareness regarding the possibility of intense exercise, such as marathon running, resulting in CRVO in young adults. Given the fact that there are currently no established guidelines for treatment in this patient population, independent case reports serve as the best available indicators for disease management. Thus, ranibizumab may be an effective treatment option for marathon runner's retinopathy.
